# Modification of a commercial DNA extraction kit for safe and rapid recovery of DNA and RNA simultaneously from soil, without the use of harmful solvents

**DOI:** 10.1016/j.mex.2015.03.007

**Published:** 2015-03-27

**Authors:** E. Tournier, L. Amenc, A.L. Pablo, E. Legname, E. Blanchart, C. Plassard, A. Robin, L. Bernard

**Affiliations:** a IRD, UMR Eco&Sols, 2 Place Viala, 34060 Montpellier Cedex 1, France; b INRA, UMR Eco&Sols, 2 Place Viala, 34060 Montpellier Cedex 1, France; c CIRAD, UMR Eco&Sols, 2 Place Viala, 34060 Montpellier Cedex 1, France; d IRD, UMR Eco&Sols-Laboratoire des RadioIsotopes (LRI), Ampandrianomby, Antananarivo 101, Madagascar

**Keywords:** Soil DNA/RNA co-extraction, Soil DNA and RNA, Quantitative co-extraction, Molecular biomass, Tropical soil

## Abstract

An optimized method, based on the coupling of two commercial kits, is described for the extraction of soil nucleic acids, with simultaneous extraction and purification of DNA and RNA following a cascade scheme and avoiding the use of harmful solvents. The protocol canmonitor the variations in the recovery yield of DNA and RNA from soils of various types.The quantitative version of the protocol was obtained by testing the starting soil quantity, the grinding parameters and the final elution volumes, in order to avoid saturation of both kits.

• A first soil-crushing step in liquid nitrogen could be added for the assessment of fungal parameters.
• The protocol was efficienton different tropical soils, including Andosol, while their high contents of clays, including poorly crystalline clays, and Fe and Al oxides usually make the nucleic acid extraction more difficult.
• The RNA recovery yield from the previous tropical soils appeared to correlate better to soil respiration than DNA, which is positively influenced by soil clay content.

## Graphical abstract

**Figure:**
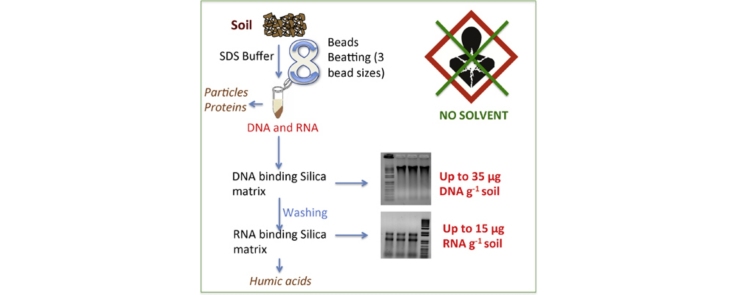

## 1 Method details

**Fig. 1:**
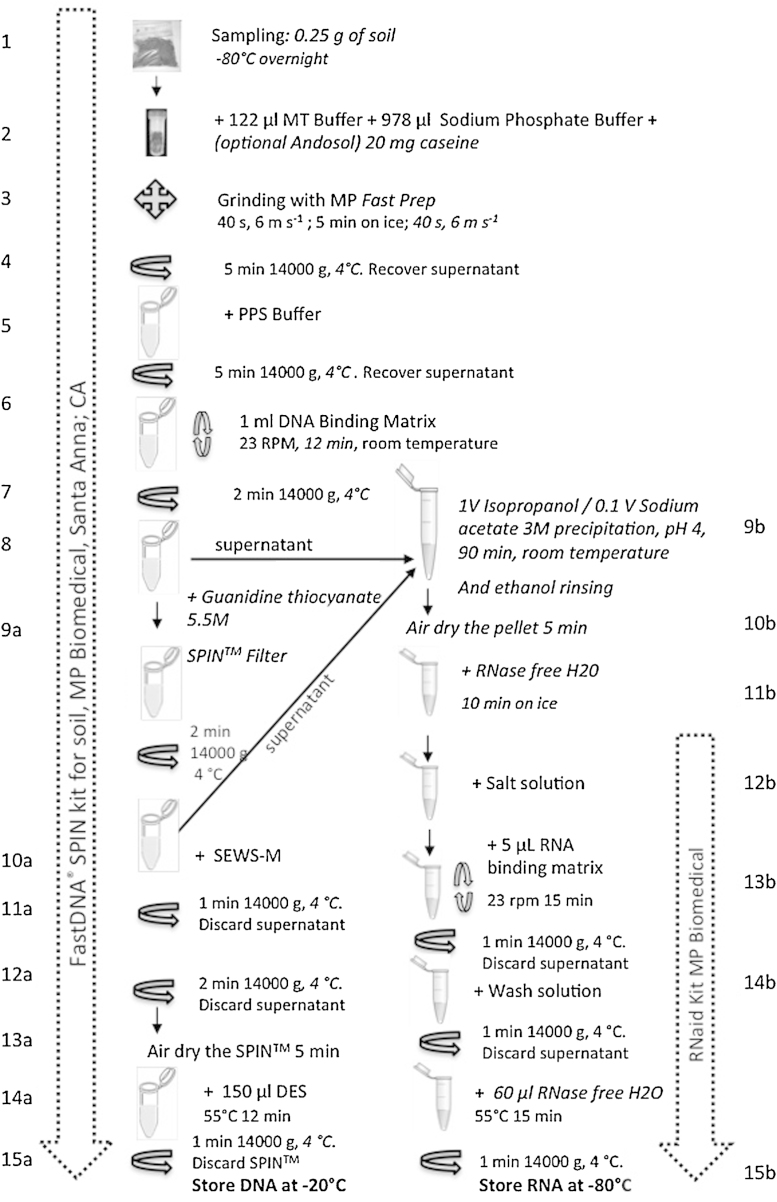
Schematic diagram of the final DNA/RNA co-extraction protocol.

We adapted a commercial DNA extraction kit (FastDNA SPIN™ kit for soil, MP Biomedical, Santa Anna, CA), to recover DNA and RNA simultaneously, avoiding the use of hazardous solvents. This DNA kit, based on an SDS extraction buffer, has already shown its efficiency [1], [2]. RNA recovery was allowed because special cares were taken to work under RNase-free conditions. All solutions and glassware were treated with diethyl pyrocarbonate (DEPC) to ensure that they were RNase free and only certified RNase- and DNase-free plastic tubes were used. Each centrifugation step was performed at 4 °C and tubes were placed on ice while waiting for the next step of the procedure. RNA could therefore be purified from the DNA washing solution at the step 8 of the protocol as shown on the flow chart presented in Fig. 1 and as further described. Modifications we brought to the initial protocol of the manufacturer are detailed *in italic character*.

**Fig. 2:**
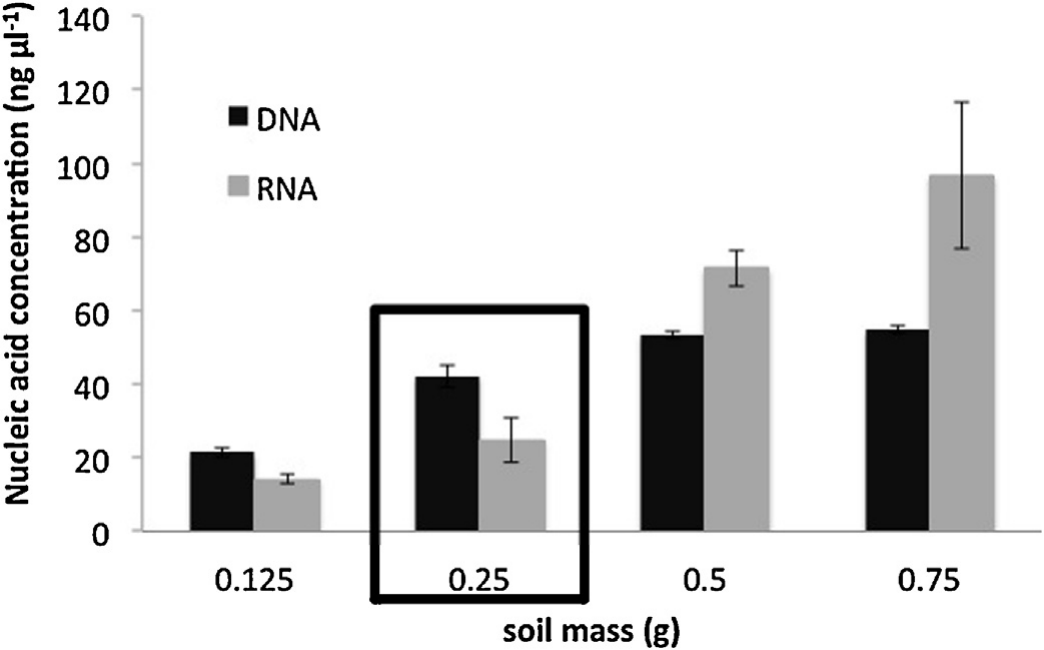
Concentrations of nucleic acids extracted (DNA and RNA) in function of the quantity of soil submitted to the extraction procedure.

**Fig. 3:**
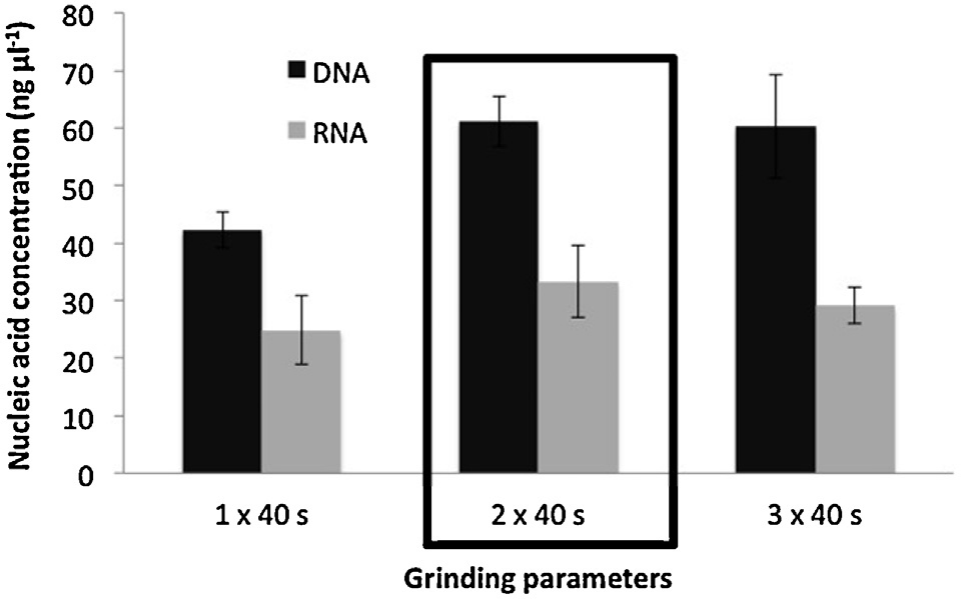
Concentrations of nucleic acids extracted (DNA and RNA) in function of the number of 40-s grinding cycles at 6 m s^−1^ performed by the FastPrep™ instrument. Errors bars correspond to 95% confidence intervals (alpha 0.05, 3 replicates). The conditions chosen as standard are framed.

**Fig. 4:**
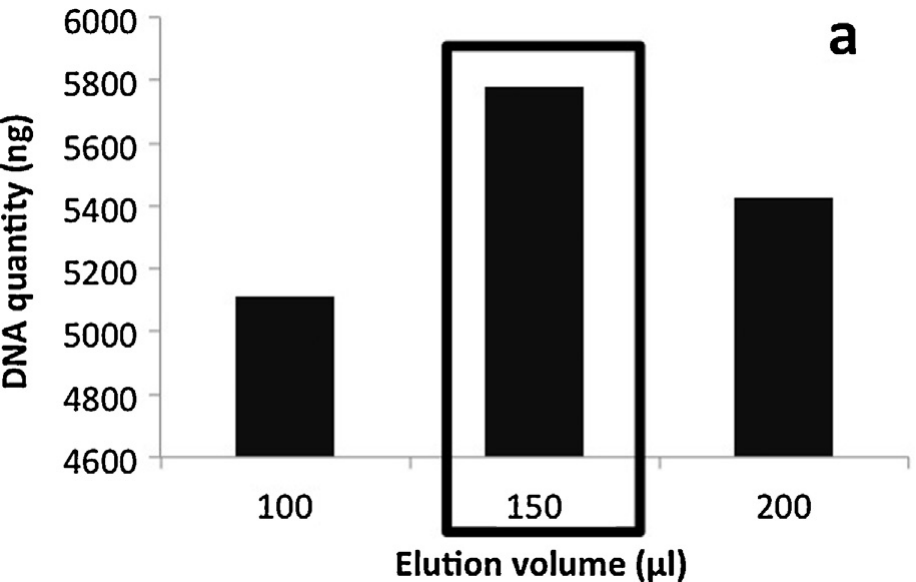
Quantity of DNA recovered as a function of the volume of elution solution. The conditions chosen as standard are framed.

**Fig. 5:**
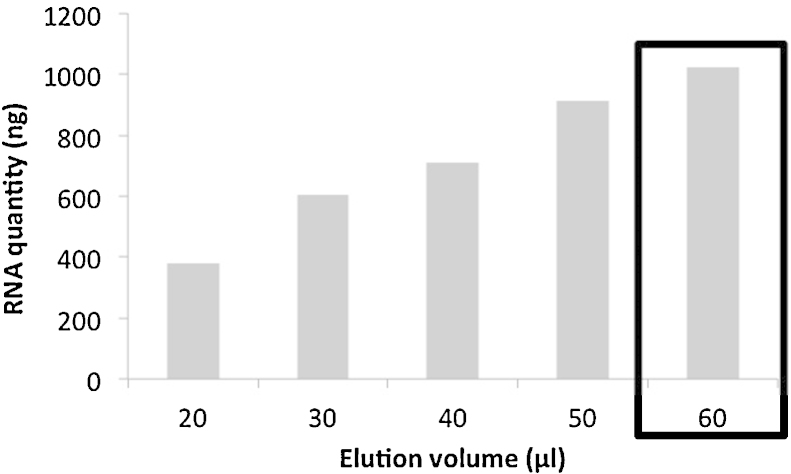
Quantity of RNA recovered as a function of the volume of elution solution. The conditions chosen as standard are framed.

1. Soil sample preparation: add 250 mg of soil sample (characteristics of soils used in this study are shown in Table 1) to a Lysing Matrix E tube and freeze overnight at −80 °C*. Initially the manufacturer preconized to use 500 mg of soil. We have tested 125, 250, 500 and 750 mg, and observed that 500 mg of soil samples led already to the maximum of DNA recovered, as 750 mg of soil did not improve the yield* (Fig. 2)*. Therefore even if 500 mg of soil increased the RNA yield by a factor of almost 4, the soil samples being processed seemed to be limited to 250 mg to keep the extracted DNA below the saturation limit of the kit and to be able to follow variations of both molecules. Of course 500 mg has to be chosen if the objective is only to maximize the quantity of DNA and RNA recovered and not to relate their recovery yield to molecular biomass estimation.*
2. Cells lysing: add 978 μL sodium phosphate buffer, 122 μL of MT buffer. *When extracting nucleic acids from andosol, 20 mg caseine has to be added to the lysing mix in order to saturate the high adsorption capacity of the non-crystallized clays* [3].
3. Grinding: homogenize in the FastPrep™ instrument (MP Biomedical) for 40 s at a speed setting of 6.0. Cool sample on ice for 5 min and perform another grinding step under similar conditions (40 s, speed 6.0). *Initially the manufacturer preconized a single homogenization step. To increase the amount of nucleic acids recovered for further analyses, a second grinding step was introduced* (Fig. 3).
4. Centrifuge at 14,000 × *g* for 5 min at 4 °C. Transfer supernatant to a clean 2.0 mL microcentrifuge tube.
5. Protein precipitation: add 250 μL PPS (protein precipitation solution) and mix by shaking the tube by hand 10 times. Keep on ice before next step, when processing other samples.
6. Centrifuge at 14,000 × *g* for 5 min at 4 °C to pellet precipitate. Transfer supernatant to a clean 2 mL microcentrifuge tube.
7. DNA binding: re-suspend Binding Matrix suspension and add 1.0 mL to supernatant in the 2 mL tube. Place on rotary shaker for 12 min, 23 rpm at room temperature, to allow binding of DNA*. Initially the manufacturer preconized a 5 min binding time but we have observed that increasing the DNA binding time from 5 to 12 min avoided DNA contamination into the washing solution containing RNA. This was verified by PCR (data not shown)*.
8. Centrifuge at 14,000 × *g* for 2 min at 4 °C to pellet DNA binding matrix. Remove the supernatant and *transfer it into a clean 15 mL tube for further RNA purification. Keep on ice before processing*.

a. DNA purification procedure:
  9 Re-suspend the DNA Binding Matrix in 500 μL of guanidine thiocyanate (5.5 M) and transfer to a SPIN™ Filter column and centrifuge at 14,000 × *g* at 4 °C for 1 min. *Transfer the eluate from the catch tube to the RNA 15 mL tube (see step 8)*.
  10 Add 500 μL prepared SEWS-M (ethanol added) and gently re-suspend the pellet using the force of the liquid from the pipet tip.
  11 Centrifuge at 14,000 × *g* at 4 °C for 1 min. Empty the catch tube and replace.
  12 Without any addition of liquid, centrifuge a second time at 14,000 × *g* for 2 minutes to “dry” the matrix of residual wash solution. Discard the catch tube and replace with a new, clean catch tube.
  13 Air dry the SPIN™ Filter for 5 min at room temperature.
  14 Gently re-suspend Binding Matrix (above the SPIN filter) in 150 μL of DES (DNase/pyrogen-free water) and incubate at 55 °C for 12 min. The final elution volume was increased compared to the initial protocol, from 100 to 150 μL to increase DNA recovery from the matrix (Fig. 4).
  15 Centrifuge at 14,000 × *g* for 1 min to bring eluted DNA into the clean catch tube. Discard the SPIN filter. DNA is now ready for downstream application.Here ended the modified protocol of the FastDNA SPIN™ kit for soil.
b. RNA purification procedure:
  9 *Add 1 volume of isopropanol and 500 μL of sodium acetate (3 M pH 4) to supernatant obtained at step 8 and 9a (usually 2.5 mL). Gently shake the tube and incubate for 90 min at −20 °C*.
  10 *Centrifuge at 14,000 × g and 4 °C for 30 min and wash the pellet with 70% ethanol and centrifuged again (14,000 *×* g at 4 °C for 5 min). Remove ethanol and air-dry the pellet for 5 min*.
  11 *Re-suspend the pellet in 100 μL of RNAase-free water and keep in ice for 10 min*.
  12 Add 300 μL of salt solution (RNaid kit-MP Biomedicals) and transfer into a new 1.5 mL tube.
  13 Add 5 μL of RNA Binding Matrix and gently shake 15 min at ambient temperature.
  14 Centrifuge at 14,000 × *g* and 4 °C for 1 min. Discard the supernatant and re- suspend the pellet in 500 μL of RNA wash solution.
  15 Centrifuge at 14,000 × *g* and 4 °C for 1 min remove the supernatant. Re-suspend the pellet in 60 μL of RNase free water, gently shake and incubate at 55 °C for 15 min. *The final elution volume was increased compared to the initial protocol from 20 to 60 μL* (Fig. 5)*. With a 60 μL elution volume, RNA yield could still be improved. However, increasing the elution volume would lead to a dilution and hence a concentration too low for subsequent reverse transcriptase reactions. Transfer the supernatant to a clean 1.5 mL RNAase free tube and keep at −80 °C prior to further application*.

## 2 Nucleic acids quantitation

**Fig. 6:**
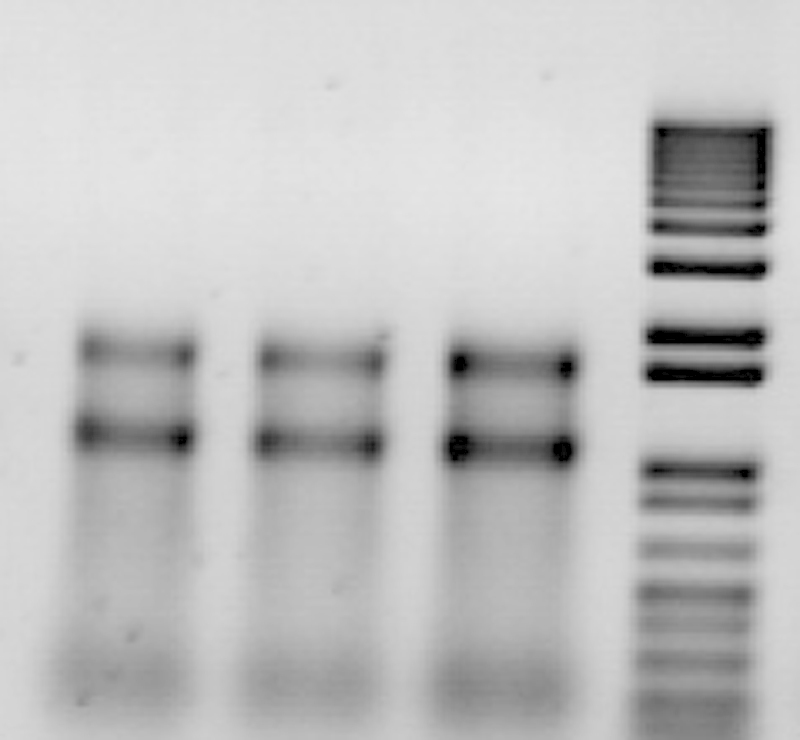
Electrophoretic profiles (agarose 1%) of 10 μL of the final RNA extract (step 15b of the protocol), and 12 μL of the 1 kb ladder (Invitrogen, France). Gel was stained with ethidium bromide.

The DNA and RNA were quantified by fluorometry using the Quant-iT™ Pico Green DNA and Quant-iT™ Ribo Green RNA assay kit (Molecular Probes, Carlsbad, New Mexico) respectively in accordance with the manufacturer’s instructions. The purity and integrity of the RNA recovered was also verified after migration on a 1% agarose gel (Fig. 6).

## 3 Option for fungal parameters analyses

**Fig. 7:**
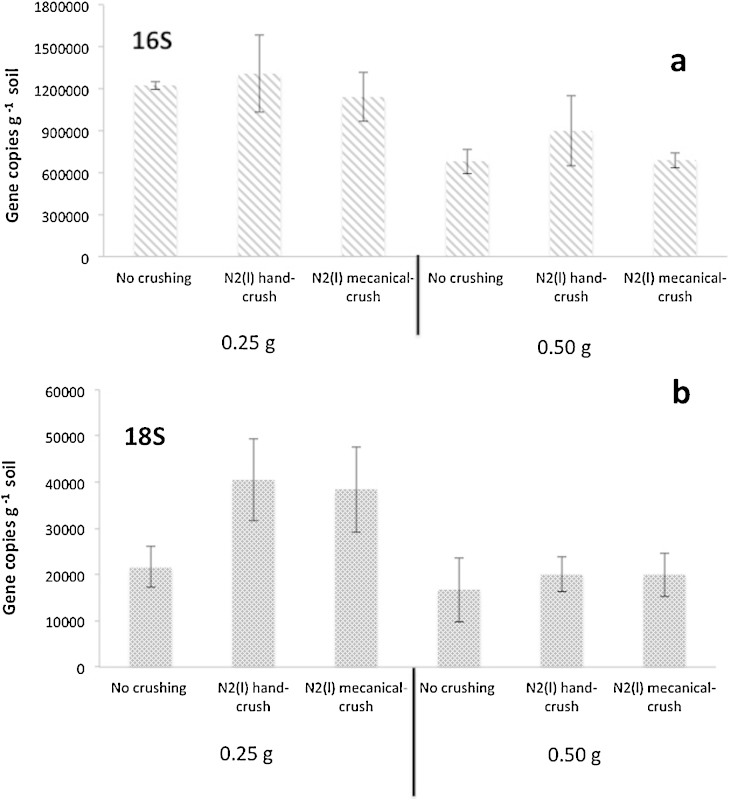
(a) Bacterial (16S) and (b) fungal (18S) ribosomal gene copy numbers per gram of soil, measured by qPCR as a function of the soil homogenization treatment before subsampling (0.25 g and 0.50 g of soil) for the co-extraction protocol. 30 g of soil were used per homogenization treatment. Errors bars correspond to 95% confidence intervals (alpha 0.05, 3 replicates).

For the analyses of fungal parameters (density and diversity), a sample size of 1 g has been recommended in order to be representative of the community [4]. Therefore, the applicability to fungal biomass quantification (see method below) was tested by crushing and homogenizing 30 g of soil in liquid nitrogen either by hand, or using a mortar grinder (Pulverisette 2, Fritsch, Idar-Oberstein, Germany) prior to subsample 0.25 g and then beginning the protocol. From our results, it appears that crushing 30 g of soil in liquid nitrogen prior to subsample 0.25 g for the further co-extraction significantly improved the recovery of 18S genes copies from the DNA while it has no effect on the 16S copy number (Fig. 7). The quantitative PCR protocol used is described in Supplementary material (S1).

## 4 Analyses of samples

**Fig. 8:**
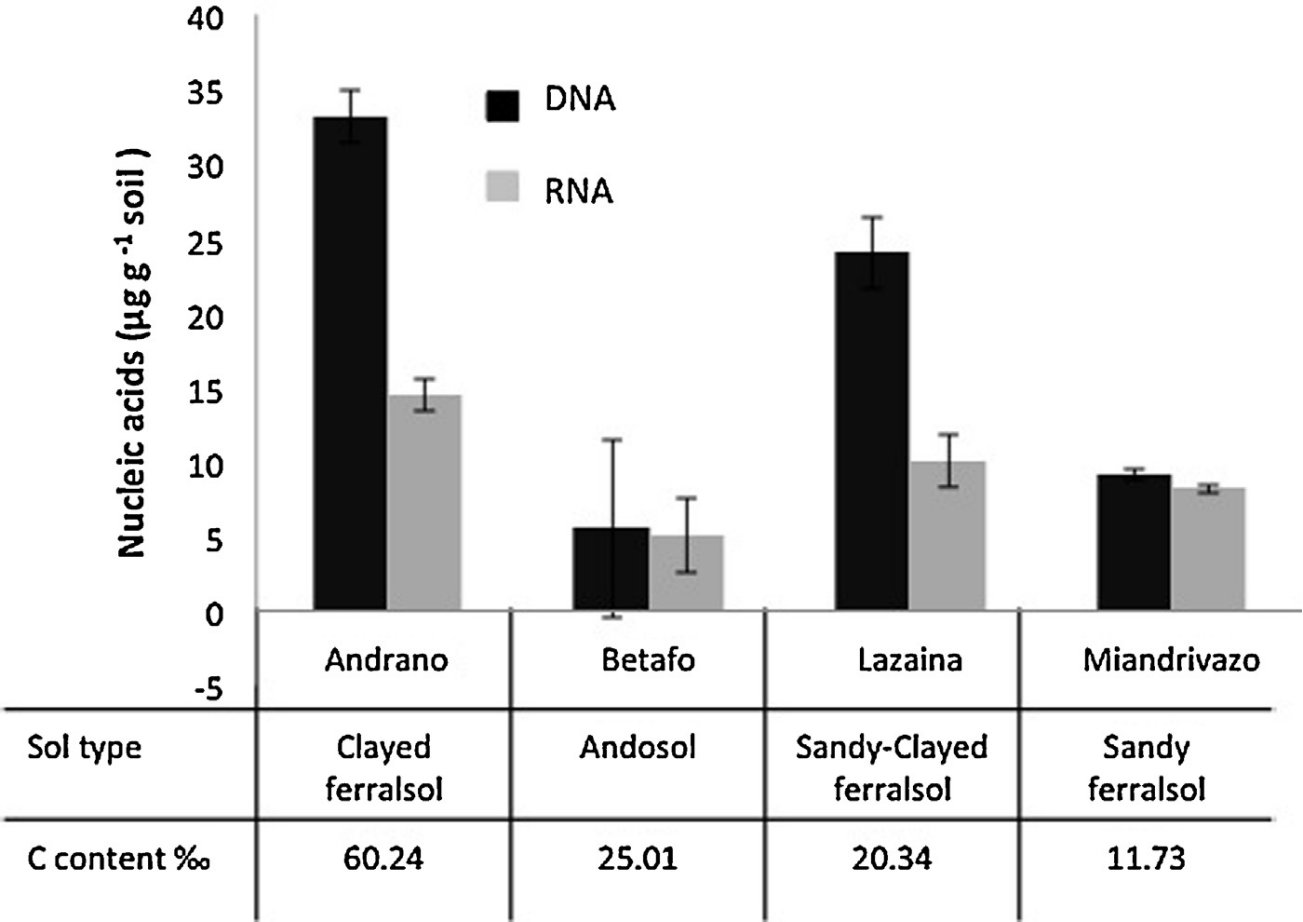
DNA (black) and RNA (light grey) extraction yields and their respective 95% confidence intervals (alpha 0.05, 3 replicates) from 4 tropical soils, sampled in Madagascar and characterized by different textures, mineralogies, metal and carbon contents. Soil types are indicated following the WRB nomenclature, and full characterization is presented in Table 1.

The co-extraction protocol was optimized on a sandy clay loam soil from South of France (Maugio – Table 1). Yields of 26.7 μg of purified DNA and 4.5 μg of purified RNA per gram of soil were obtained, with a very good repeatability for both DNA and RNA (coefficients of variation of 8.69% for DNA and 5.25% for RNA when applied to a set of 10 replicated soil samples). The protocol was tested on 4 tropical soils from Madagascar, which were chosen because their composition was thought likely to complicate the extraction of nucleic acids (Table 1). One major problem is the presence of minerals known to adsorb organic molecules strongly, namely clays (Andranomanelatra, Betafo and Lazaina) and metal oxides (all soils), and especially when clays are poorly crystallized (Betafo and to a lesser extend Andranomanelatra). Another problem is the small size of the microbial biomass pool, which is usually characteristic of carbon depleted sandy soil (Miandrivazo). The most difficult soil was the Andosol containing allophanes (Betafo), which required the addition of 20 mg caseine to the extraction mix just before the FastPrep grinding step (protocol step 2). The four soils were extractable and gave various DNA and RNA recovery yields sufficiently concentrated to be further analyzed by any other molecular technique (Fig. 8). As expected, the mean RNA/DNA ratio observed for tropical soils (0.62) was greater than that for the Mediterranean soil sampled during the winter-time (0.17).

## 5 Additional information

Variations in the DNA and RNA composition bring additional information as DNA is linked to cell replication while RNA is linked to protein synthesis. A comparison of the composition of microbial communities based on co-extraction of both DNA and RNA can provide an insight into the ecology of populations, provided that DNA and RNA are subjected to the same extraction bias. However, to date, very few studies developed methods for DNA/RNA co-extraction from soil samples [5], [6], [7], [8], [9], [10]. With the exception of the method described by [8], all procedures have required harmful solvents such as phenol, chloroform and β-mercaptoethanol, which are carcinogenic, mutagenic reproductive toxins. The DNA/RNA co-extraction protocol developed in the present work used DEPC and guanidine thiocyanate, which are just irritants by contact or ingestion. SDS-based methods are thought to be less efficient than solvent-based methods. Using a solvent-based method, [5] obtained yields between 10 and 20 μg of DNA and 2 and 5 μg RNA per gram of soil, in an unpurified extract. In order to purify such extracts, the sample would have to be divided into two aliquots and each one digested with either DNAse or RNAse thus decreasing the yield of both RNA and DNA.

**Fig. 9:**
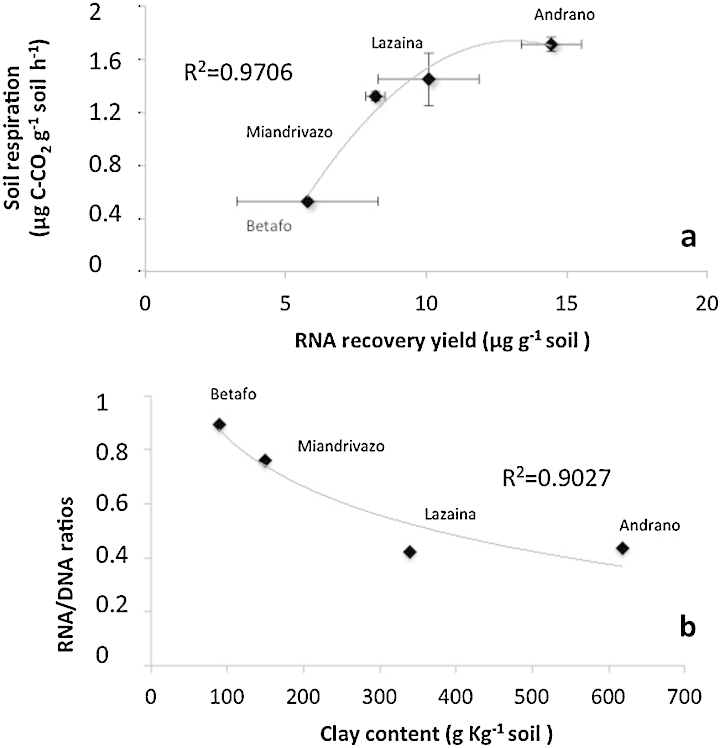
XY dot plots of (a) soil respiration against RNA extraction yields and their respective 95% confidence intervals (alpha 0.05, 3 replicates), and (b) RNA/DNA against soil clay content. Best fitted relationships followed second order polynomial functions. Regression coefficients *R*^2^ are indicated.

Recently, the quantity of DNA extracted from soil had been defined as “microbial molecular biomass” and proposed as a microbial indicator of soil status [11], [12], [13], [14]. This notion of considering total recovered DNA as a total biomass quantification tool is still strongly debated in microbial ecology because DNA can remain stable in the environment for long periods of time. In their DNA extraction protocol, [14] added a first step of soil washing to first desorb the extracellular DNA from the mineral matrix [15]. Such step can also be added into the present protocol. But the particularity of the present method is to simultaneously co-extract RNA, which is a much more labile molecule than DNA. On the four precedent soils from Madagascar, we have measured the CO_2_ evolution (soil respiration) at pF 2.5 during 6 h at 25 °C. When plotted the respiration against DNA and RNA recovery yields we found a much better correlation with RNA (Fig. 9a; R2: 0.97) than with DNA (data not shown; R2: 0.66). This can be explained by the high affinity of extracellular DNA for clays as RNA/DNA ratio inversely correlated to clay content (Fig. 9b). Therefore RNA recovery yield should be a better indicator of living microbial biomass than DNA. Now this protocol has been developed and tested, future studies will be conducted to determine whether the RNA/DNA ratio of the microbial communities is a reliable bioindicator of soil status in a context of global changes (climate, land uses, agricultural practices…), provided that such effects are studied on the same soil or soils with similar clay content.

Future technical optimizations could include the recovery of messenger RNA to quantify the expression of functional genes, compared to their presence into the DNA fraction, as well as the recovery of enzymes from the precipitate obtained at step 5 as we already verified that PPS does not inactivate them (data not shown).

## Figures and Tables

**Table 1:** Characterization of the soils used to develop and to test the DNA/RNA co-extraction method.

Soil	Maugio	Andranomanelatra	Betafo	Lazaina	Miandrivazo
GPS ordination	35°9′03″E, 43°7′14″N	19°46′42.19″S, 47°06′28.70″E	19°50′05.92″S, 46°50′35.49″E	18°46′54.45″S, 47°32′05.99″E	19°32′51.15″S, 45°28′08.43″E
Water pH	8.29	4.42	6.25	6.14	6.55
Clay, g (kg soil)^−1^	248	619	149	340	90
Silt, g (kg soil)^−1^	436	188	469	70	194
Sand, g (kg soil)^−1^	350	193	382	580	716
Al_DCB_^a^, g (kg soil)^−1^	ND	19.9	28.7	7.2	1.44
Fe_DCB_^a^, g (kg soil)^−1^	ND	62.1	60.3	35.13	8.61
Si_DCB_^a^, g (kg soil)^−1^	ND	4.2	1.3	0.98	4.15
Al_ox_^b^, g (kg soil)^−1^	ND	17.8	34.6	2.95	1.48
Fe_ox_^b^, g (kg soil)^−1^	ND	4.5	24.3	1.41	1.77
Si_ox_^b^, g (kg soil)^−1^	ND	1.9	15.9	0.24	0.73
Al_p_	ND	5.72	6.78	3.04	0.29
Fe_p_	ND	3.04	2.06	1.73	0.23
Si_p_	ND	<0.01	0.34	0.72	0.73
Carbon, g (kg soil)^−1^	9.30	60.24	25.00	20.34	11.73
C/N	10.40	12.73	13.15	14.95	11.61
CEC cmol (kg soil)^−1^	21.10	17.32	29.59	6.20	14.25

ND: not determined.
