# Supplementary material for: Modification of a commercial DNA extraction kit for safe and rapid recovery of DNA and RNA simultaneously from soil, without the use of harmful solvents
Source: MethodsX. 2015 Mar 27;2:182–91. doi: 10.1016/j.mex.2015.03.007 (PMC4487712; doi:10.1016/j.mex.2015.03.007)
Supplement: Supplementary file 1 [file mmc1.docx]

Gene copy quantitation

Bacterial (16S) and fungal (18S) ribosomal gene copy numbers per gram of soil were measured by qPCR technique in function of the soil homogenization treatment before subsampling (0.25 g and 0.50 g of soil) for the co-extraction protocol. The 10 µL volume of amplification mix was composed of 10 ng of extracted DNA, 5 μl of the Absolute SYBR green® (2x) PCR master mix (Thermo Fisher Scientific, Waltham, Massachusetts), 250 ng of T4gp32 (MP biomedical) and 1 (16S) or 1.25 (18S) µM of each primer (16S: 341F-CCTACGGGAGGCAGCAG and 515R-ATTACCGCGGCTGCTGGCA [15]; 18S: FR1- AICCATTCAATCGGTAIT and FF390-CGATAACGAACGAGACCT [16] and [17]. Reactions were performed using a CFX 96 (BIORAD, Marnes-la-Coquette, France) following 16S and 18S specific programs (16S: 95°C [15min]-1 cycle, 95°C [15s], 60°C [30s], 72°c[30] - 35 cycles; 18S : 95°c [15min]-1 cycle, 95°C[15s], 50°C[30s] 72°C [30s] - 40 cycles).
